# Editorial: Aging, lifestyle, and cellular health

**DOI:** 10.3389/fnut.2025.1578242

**Published:** 2025-04-09

**Authors:** Leonidas G. Karagounis, Amira Kassis

**Affiliations:** ^1^Mary MacKillop Institute for Health Research, Australian Catholic University, Melbourne, VIC, Australia; ^2^Institute of Social and Preventive Medicine, University of Bern, Bern, Switzerland; ^3^Neat Science, Chatel Saint-Denis, Switzerland

**Keywords:** aging, nutrition, lifestyle, health, bioactives, cellular health

By 2050, the global population of adults aged 65 and older will exceed 1.6 billion, constituting 1 in 6 people, up from 1 in 11 in 2019 (1). Although life expectancy has risen, health span has not kept pace, leading many to experience a decade of poor health in later life. Population aging has profound economic implications, including workforce contraction, reduced tax revenue, increased demand for government support programs, and escalating healthcare costs, straining government budgets.

Aging is an inevitable biological process driven by molecular mechanisms, accumulated life behaviors, and diminished physiological plasticity. These factors collectively accelerate functional decline and elevate the risk of age-related diseases. Understanding aging requires analyzing both system-specific changes and inter-system interactions. The immune system, for instance, undergoes immunosenescence, inflammaging, and dysbiosis, weakening defenses against infections (2). In the cardiovascular system, vascular aging impairs endothelial function and vasodilation, increasing cardiovascular disease (CVD) risk (3). Metabolic dysfunction, involving nutrient sensing disruptions, chronic inflammation, and oxidative stress, exacerbates atherosclerosis and arterial stiffness, further elevating cardiovascular risk. Parallel degenerative changes in the nervous and musculoskeletal systems contribute to cognitive decline and sarcopenia (4). Furthermore, accelerated aging in one organ, impacts others through shared pathways involving inflammation, oxidative stress, and hormonal dysregulation, leading to comorbidities. This concept of multiorgan aging underscores the complexity of age-related diseases.

Diet is known to significantly influence the promotion of both health and disease. Research has traditionally focused on macronutrients, micronutrients, and trace elements, yet food metabolism and its bioactive compounds are increasingly recognized as critical to aging. The USDA National Nutrient Database catalogs 7,793 food items and 150 components, a fraction of the 26,000 known food-derived biochemicals (5). Additionally, microbial metabolism generates over 44,000 compounds, many with biological functions (5). This Research Topic highlights the impact of bioactive compounds on cellular health and longevity, particularly those targeting microbiome-related and inflammatory mechanisms affecting specific biological functions.

The manuscripts included in this collection showcase the emerging evidence on nutritional bioactives and their impact on hallmarks of aging, emphasizing gut microbiota's role in health, notably cognitive and muscle health. Dysbiosis contributes to intestinal barrier dysfunction, harmful metabolite release, and negative effects on muscle and neural tissues. Gut microbiota also regulate immune responses, neurotransmitters, and metabolites, influencing muscle and cognitive function. Age-related muscle loss and cognitive decline correlate with inflammation, oxidative stress, and microbiota composition, driving interest in preventive and therapeutic strategies.

A study by Cai et al. found that 12-week oral administration of *Lacticaseibacillus paracasei* (LC86) alleviated sarcopenia and cognitive decline in senescence-accelerated mouse prone 8 (SAMP8) mice (Cai et al.). Treatment improved health span, reduced aging phenotypes, increased muscle glycogen reserves, enhanced muscle strength, and improved cognitive performance. LC86 also modulated neurotransmitter levels, reduced systemic inflammation, enhanced hepatic antioxidant defenses, and promoted beneficial gut microbiota shifts.

Tryptophan, an essential amino acid, serves as a precursor for metabolites and neurotransmitters while regulating inflammation. Malnutrition-associated tryptophan depletion increases vulnerability in patients. Ritz et al. analyzed a clinical trial subset to evaluate tryptophan pathway metabolites' prognostic significance. Malnourished patients had lower tryptophan levels, correlating with disease severity but not nutritional intake or risk scores (NRS 2002) (Ritz et al.). Low tryptophan, kynurenine, and serotonin levels predicted higher 30-day and 180-day mortality rates. Despite increased mortality risk, nutritional support was less effective in these patients. The study concluded that tryptophan and its metabolites provide independent prognostic value in at-risk individuals (Ritz et al.).

Aging induces skeletal muscle changes, including fiber loss and atrophy, reducing strength. Muscle mass remains stable until approximately age 40, with accelerated declines after 65–70 years. Aging muscles also exhibit disrupted protein expression, mitochondrial abnormalities, impaired ion regulation, and altered stress responses. Eggshell membrane (ESM) is rich in bioactive compounds, including proteins, glycosaminoglycans (GAGs), and N-glycans, known for promoting joint health, wound healing, and skin integrity in clinical and *in vitro* studies. ESM hydrolysates retain anti-inflammatory properties post-digestion and may alleviate joint pain and stiffness. ESM powder has demonstrated efficacy in reducing intestinal inflammation and microbial dysbiosis while enhancing skeletal muscle mass (Rønning et al.), although its molecular effects on muscle remain underexplored. Specifically, recent findings indicate that ESM intake in elderly mice mitigates aging hallmarks, including muscle fiber loss, altered fiber composition, and gene expression changes related to muscle atrophy and regeneration (Rønning et al.). It also promoted a younger muscle phenotype at the protein level. ESM consumption increased gut microbiota diversity, altered composition, and reduced inflammation markers in mice and human studies (Rønning et al.). These findings suggest ESM as a potential nutraceutical for counteracting skeletal muscle aging via immunomodulation and microbiota interactions, though further research is warranted.

Finally, in their comprehensive review, Jacquier et al. evaluated the effects of phytonutrients on cellular, organ, and functional aging parameters using a framework cantered on vitality, intrinsic capacity, and expressed capacities in older adults. The review proposes a paradigm shift toward including specific phytonutrients in a preventive nutritional strategy based on their capacity to modulate specific hallmarks of aging.

Aging is a multifaceted process affecting multiple organ systems, leading to physiological decline and increased disease risk. Although life expectancy has improved, extending health span remains a challenge. Emerging research underscores the role of nutrition, gut microbiota, and bioactive compounds and how the cohesive interaction of these parameters may modulate aging by preserving functional health. Dietary interventions and nutraceuticals may provide innovative solutions for promoting healthy aging and reducing age-related disease burden. However, further studies are necessary to translate these findings into practical applications for extending health span and improving quality of life.
